# Thoracic Myelopathy in a Teenager Caused by Stage IV Hodgkin’s Lymphoma

**DOI:** 10.7759/cureus.38298

**Published:** 2023-04-29

**Authors:** Kennedy A Forest, Allison M Hemmer, Kelly Stacy, MD

**Affiliations:** 1 Internal Medicine, University of South Dakota Sanford School of Medicine, Vermillion, USA; 2 Internal Medicine, Monument Health, Rapid City, USA

**Keywords:** hodgkin’s lymphoma, health disparities and vulnerable populations, thoracic myelopathy, covid 19, native american

## Abstract

We report the case of a 19-year-old Native American woman who presented with bilateral lower extremity weakness due to spinal cord compression from late-stage Hodgkin's lymphoma. Hodgkin's lymphoma rarely has an initial presentation of spinal cord compression, except in cases of late-stage disease. The patient partially attributed her delayed pursuit of care to the difficulty of scheduling an appointment during the coronavirus (COVID-19) pandemic. The COVID-19 pandemic has impacted access to care and the potential for early detection of disease, as seen in this patient. Additionally, Native Americans on South Dakota Reservations face unique challenges that affect access to healthcare and health outcomes.

## Introduction

Spinal cord compression (SCC) from Hodgkin's lymphoma (HL) causing myelopathy is rarely the presenting symptom of the disease. The usual presentation of Hodgkin’s lymphoma includes constitutional symptoms and painless lymphadenopathy [1,2]. Reported cases where SCC is the initial presentation is often a sign of late-stage disease [3]. We present a unique case where early detection of disease may have been missed due to foregoing medical assessment during the COVID-19 pandemic, as well as the impact of other disparities in access to care for Native American populations in South Dakota.

## Case presentation

**Figure 1:**
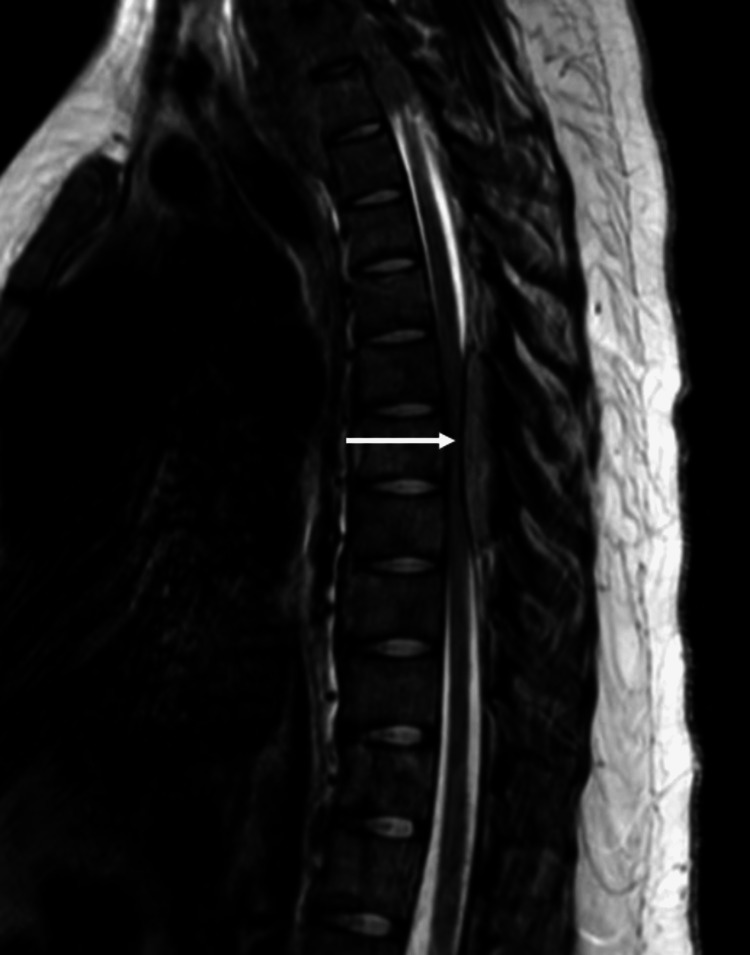
Sagittal T2 STIR MRI of the thoracic spine. MRI of the thoracic spine revealed a soft tissue tumor (white arrow) in the spinal canal at the T6-T9 level compressing the mid-thoracic spinal cord. STIR: short inversion time inversion recovery

We report the case of a 19-year-old Native American woman who presented to the emergency department with bilateral lower extremity weakness. She reported progressive leg weakness for one week, causing loss of balance and multiple falls. She was non-ambulatory at the time of her presentation. She suffered intermittent back pain for two months preceding the leg weakness. At the examination, she was febrile (39.6°C) and tachycardic (116 bpm) with normal blood pressure. Neurological examination revealed bilateral lower extremity weakness with the inability to lift her legs from the bed with extended knees and decreased motor function of the ankles, feet, and toes bilaterally. The sensation was impaired bilaterally in her lower extremities. A complete blood count revealed microcytic anemia with a hemoglobin of 7.6 g/dL (reference range 11.5-15.5), a mean corpuscular volume of 77.6 fL (reference range 81-97), and leukocytosis with a white blood cell count of 15.3 10*3/uL (reference range 4.5-10.5*3). Spinal computed tomography (CT) revealed masses along the cervical and thoracic spines. An epidural thoracic 5-8 posterior lesion, as seen in Figure 1, was revealed with severe spinal stenosis causing thoracic myelopathy.

A CT scan of the chest, abdomen, and pelvis revealed extensive metastatic disease with "too numerous to count" bilateral pulmonary nodules. There were diffuse mediastinal and axillary retroperitoneal lymph node enlargements. There were extensive sclerotic and lytic bony metastases throughout the visualized skeleton.

**Figure 2:**
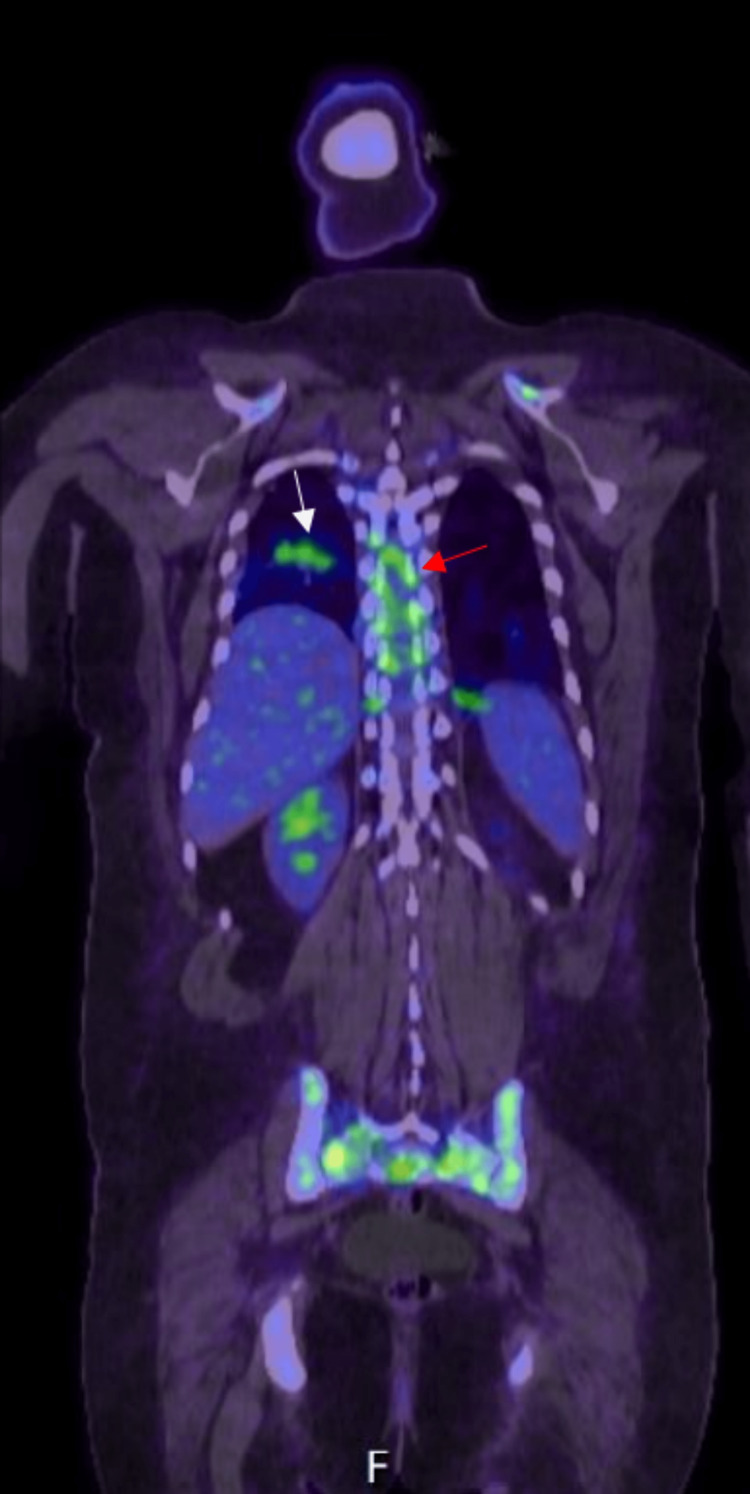
Coronal fused PET-CT skull base to mid-thigh. The PET scan revealed multiple hypermetabolic pulmonary masses (white arrow) compatible with lymphoma and multiple hypermetabolic osseous lesions compatible with malignant involvement, including multiple vertebrae (red arrow), sacrum, and pelvis.

A spinal MRI revealed diffuse osseous metastatic disease with the extension of the soft tissue tumor beyond the bony cortices and a paraspinal soft tissue mass extending inferiorly from C3. Prominent soft tissue masses in the ventral and lateral spinal canals at the T1 and T2 levels were present. A soft tissue tumor in the spinal canal at T6-T9 level compressed the mid-thoracic spinal cord seen in Figure 1. A brain MRI revealed no apparent intracranial metastases. The extent of disease seen on a positron emission tomography (PET) scan can be observed in Figure 2.

An emergent T6-T9 decompressive laminectomy was performed with central decompression and lateral recess decompression with the removal of extradural spinal lesions. The immunohistopathological study of the thoracic lesions revealed classical Hodgkin’s lymphoma, nodular sclerosing type. Based on the extent of the disease's spread, she was determined to be at stage IV. The recommended treatment was discussed with the patient. Her initial treatment plan consisted of doxorubicin, bleomycin, vinblastine, and dacarbazine (ABVD) chemotherapy for two cycles, followed by PET restaging based on the Deauville Criteria score to determine the next steps in treatment.

In the days following the laminectomy, the patient had a slight improvement in sensory and motor function in her lower extremities. Physical therapy and occupational therapy were initiated, and her symptoms continued to slowly improve. After one cycle of ABVD, she developed pulmonary complications and was switched to chemotherapy utilizing brentuximab vedotin in place of bleomycin (A+AVD). After two cycles of chemotherapy, PET restaging showed a mixed response with interval resolution in certain areas as well as new areas of increased uptake. The plan was to complete six total chemotherapy cycles and then complete restaging via PET scan. Unfortunately, the patient was lost to follow-up prior to completing the recommended chemotherapy cycles.

## Discussion

Hodgkin's lymphoma (HL) is a hematologic malignancy involving the lymph nodes. It is estimated there are around 8,400 new cases of HL in the United States each year [1]. It tends to have a bimodal age distribution, first peaking between the ages of 15 and 30 and again in the sixth decade [2]. Spinal cord compression (SCC) is not common and develops in 5% of total cases; it is the initial presentation of disease in only 0.2% of cases. When present in HL, it tends to be a late manifestation of the disease or occurs during relapse. SCC is nearly three times more common in non-Hodgkin's lymphoma than in HL [3].

HL is typically a disease of the lymph nodes, but extranodal sites may be involved in up to 10% of cases [1,2]. The spleen, liver, lungs, and bone marrow are the most commonly involved extranodal sites [1]. It is hypothesized that epidural masses that lead to SCC may arise from hematogenous dissemination or direct spread from thoracic or retroperitoneal lymph nodes [3]. The thoracic segment of the spinal cord seems to be most frequently affected, followed by lumbar and cervical involvement [4].

At the time of diagnosis, patients typically have painless supradiaphragmatic lymphadenopathy, most commonly involving the cervical lymph nodes. Night sweats, fever, weight loss, and chronic pruritus may also be present [1,3,4]. Rarely, HL may manifest as back pain with or without neurological abnormalities if the spinal cord is being compressed due to an epidural mass [3,4].

**Figure 3:**
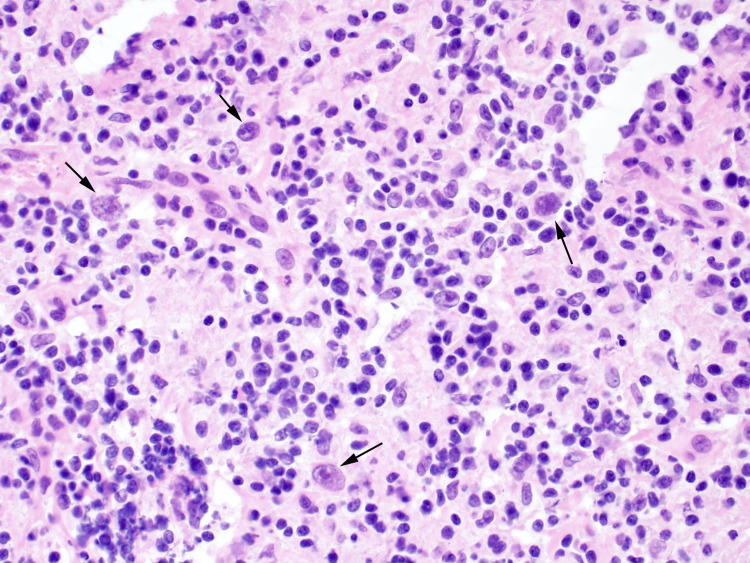
Lymph node with Hodgkin's lymphoma Reed-Sternberg cells. Lymph node with scattered Reed-Sternberg cells (arrows) with prominent nucleoli and abundant cytoplasm diagnostic of Hodgkin's lymphoma [6].

The initial diagnosis of HL is made by an excisional lymph node biopsy. Fine needle aspiration or core needle biopsies are inadequate as they do not capture the entire architecture of the node, which could potentially leave malignant cells behind. As seen in Figure 3, confirmation is made by identifying malignant Reed-Sternberg cells [3]. A PET scan will be helpful to determine the full extent of disease spread and assist in staging [2,5].

The current gold standard treatment for HL is an ABVD chemotherapy regimen consisting of doxorubicin, bleomycin, vinblastine, and dacarbazine [1,2]. Most recent research (published after the initial treatment of the patient in this report) suggests a potential survival benefit for the treatment of stage III or IV HL when utilizing brentuximab vedotin instead of bleomycin, a treatment known as A+AVD [7]. Radiotherapy is often used in conjunction with a patient's chemotherapy regimen. In patients with SCC, surgical decompression may be indicated for relief of compression and to allow for tissue sampling [1,2]. Though SCC in HL is rare, it must be identified and treated promptly as an oncologic emergency due to impending neurologic effects and mortality [8].

The prognosis depends on several factors, including the clinical stage of the disease. For those in stages I or II, the five-year overall survival is nearly 90%, whereas, for those in stage IV, the five-year overall survival is around 60%. HL involving the epidural space tends to have a relatively good prognosis if treated promptly and adequately. Around 85% regain neurologic function, and 61% have a complete clinical response [9].

This patient shared that months prior to her presentation in the emergency department, she had scheduled an appointment for evaluation of a persistent, painless cervical "lump". That appointment was canceled due to the COVID-19 pandemic. There have been many disruptions and delays in medical care since the COVID-19 pandemic began. It is estimated that nearly 41% of US adults postponed or avoided medical care due to concerns about the pandemic [10,11]; 22.3% of South Dakota households had children who missed, delayed, or skipped preventative checkups due to the pandemic [12]. Although there is no way to predict whether the canceled appointment for this patient would have led to an earlier lymphoma diagnosis, delayed access to medical care during this time has been shown to increase morbidity and mortality for acute and chronic health conditions that were unable to be addressed [10,11].

In this case, the patient was a Native American who lived on a South Dakota Native American Reservation. Unique disparities that Native American communities face in accessing healthcare include low funding for Indian Health Services (IHS) and tribal facilities, communication issues between patients and providers, logistical complications (transportation, family care, finances), and a perceived lack of emotional support and trust in the healthcare systems [13,14]. These disparities create barriers when seeking care that plays a role in the early detection of disease and obtaining appropriate care in a timely manner. Data suggests that when compared with patients from other races, Native American patients with cancer are more likely to present with late-stage disease (stage III or stage IV). Ultimately, this leads to lower survival rates for this population [13,14].

## Conclusions

As seen in the presentation of this patient, spinal cord compression causing myelopathy can be an outcome of late-stage Hodgkin’s lymphoma in rare cases. The COVID-19 pandemic impacted timely access to care around the world. Physicians and patients will likely continue to witness the consequences of delayed detection of disease due to the pandemic. The challenges of the pandemic as well as the barriers that Native Americans face when seeking access to healthcare seem to contribute to the late presentation of diseases, as seen in this patient.
